# Secular Trends in an Indian Intensive Care Unit-database Derived Epidemiology: The Stride Study

**DOI:** 10.5005/jp-journals-10071-23175

**Published:** 2019-06

**Authors:** Manu Varma MK, Bhuvana Krishna, Sriram Sampath

**Affiliations:** 1-3 Department of Critical Care Medicine, St. John's Medical College and Hospital, Bengaluru, Karnataka, India

**Keywords:** Customized health in intensive care trainable research and analysis tool (CHITRA), International Statistical Classification of Diseases and Related Health Problems 10th Revision (ICD 10), Admission diagnosis, Comorbidity, Intensive care unit (ICU)

## Abstract

**Context:**

The Indian Society of Critical Care Medicine (ISCCM), had taken an initiative to enable all Indian ICUs (Intensive Care Unit) to capture and store relevant data in a systematic manner in an electronic database: “CHITRA” (Customized Health in Intensive Care Trainable Research and Analysis tool).

**Aims:**

This study was aimed at capturing, and summarising longitudinal epidemiological data from a single tertiary care hospital ICU (Intensive Care Unit), based on a pre-existing database and the CHITRA system.

**Settings and design:**

Prospective Observational

**Materials and methods:**

Data was extracted from two databases, a pre-existing database, arbitrarily named pre-CHITRA (January 2006 to April 2014), and the CHITRATM database (October 2015 to January 2018). Diagnoses of the patients admitted were tabulated using the ICD10 (International Statistical Classification of Diseases and Related Health Problems 10th Revision) coding format. The outcomes were summarised and cross tabulated.

**Statistical analysis used:**

Cross tabulations were used to display summarized data, analysis of outcomes were done using t test and regression analyses, and correspondence analysis was used to explore associations of descriptors.

**Results:**

A total of 18940 patients were admitted, with a male preponderance, and the median age was fifty-two years. Most of admissions were from emergency (62%). The age (0.3, *p* = 0.000, CI (0.2 - 0.38)) and mean APACHE II score of patients had increased over the years (0.18, *p* = 0.000 CI (0.12-0.25). The ICU mortality had decreased significantly over the years (–0.04, *p* = 0.000, CI (–0.05 to –0.03)). The most common admission diagnosis in the pre-CHITRA database was general symptoms and signs (ICD10 R50-R69), and in the CHITRA database was Type1 Respiratory failure (ICD 10 J96.90).

**Conclusion:**

This study has shown the utility of the CHITRA system in capturing epidemiological data from a single centre.

**Key messages:**

The utility of the CHITRA system in capturing epidemiological data has been shown.

**How to cite this article:**

Manu Varma MK, Krishna B, Sampath S. Secular Trends in an Indian Intensive Care Unit-Database Derived Epidemiology: The Stride Study. Indian J Crit Care Med 2019;23(6):251–257.

## INTRODUCTION

The practice and services provided by intensive care units (ICUs) vary across and within the countries.^1^ Patients in different ICUs can exhibit substantial variations in comorbidities, case-mix, and severity of illness scores. In ICUs, secular variations in patient population and severity of illness are not unexpected.^2,3^ Longitudinal epidemiological studies can be utilized to assess and quantify the impact of changes in healthcare delivery.^4^

Studies have compared mortality among gender and different age groups.^4,5^ A time series analysis in United States ICU from 1988 to 2012 showed mortality reduction accompanied by an increase in severity of illness and chronic health conditions.^6^ Current available epidemiological studies from Indian ICUs are restricted to multicentre cross-sectional studies or descriptive studies pertaining to specific subgroups of patients.^7,8^

In Indian ICUs, organized data collection and analysis are at different stages of development.^9^ Current utilization of computing technology in Indian ICUs are limited to the most basic technology, such as access to laboratory data or medical imaging.^10^ The private healthcare sector in India has a limited form of electronic health records (EHR) but has not begun public exchange of health information to improve quality of care; however, the situation is changing due to the efforts being made by government and industry.^11^

In the year 2014, Indian Society of Critical Care Medicine (ISCCM), took an initiative to enable all Indian ICUs to capture and store relevant data in a systematic manner in an electronic data base “CHITRA” (Customized Health in Intensive Care Trainable Research and Analysis tool).^12^ This database did not require a sophisticated EHR, and aimed to be an independent system.^12^

The current study is aimed at capturing and summarizing longitudinal epidemiological data from a single tertiary care hospital ICU, based on a preexisting database and the CHITRA system.

## SUBJECTS AND METHODS

The study was performed in a 30-bedded tertiary care ICU of 1,200 beds medical college hospital. Approval was obtained from the Institutional Ethics Committee (IEC; Ref No. 237/2015). Informed consent was obtained from the legally authorized representative at admission.

Data were extracted from two databases, a preexisting database and the CHITRA^TM^ database. The preexisting database had information of patients who were admitted to the ICU between January 2006 and April 2014. This database will henceforth be referred to as pre-CHITRA database in this article. Patients admitted to the ICU between 1st October 2015 and 1st January 2018 had data captured at bedside computers in which the CHITRA database had been installed. Material for this study was extracted from both the databases. Admitting diagnoses of each patient was classified as comorbidities or disease by the medical staff and was entered into CHITRA database. Data necessary for calculating severity of illness was captured by ICU secretarial staff who had been trained for data entry. At discharge, outcomes were captured by secretarial and senior nursing staff. Formal training was given to medical and nursing staff to ensure that data entry was part of their daily work pattern. Random checks were done to ensure completeness and accuracy of data capture.

The diagnoses of the patients admitted from year 2006 to 2014 were extracted from pre-CHITRA database and tabulated using the ICD10 (International Statistical Classification of Diseases and Related Health Problems 10th Revision) coding format.^13^ Admission diagnoses entered in the pre-CHITRA database had been stored as ICD9 codes, which are now obsolete. These ICD9 codes were then converted to current ICD10 codes using mapping tools provided by the Unified Medical Language System (UMLS) terminology services of the U.S National Library of Medicine, National Institutes of Health.^14^ The CHITRA database automatically generates and stores the data as SNOMED CT^TM^ (Systematized Nomenclature of Medical Terms, Clinical Terms http://www.snomed.org) codes from the entered variables. To facilitate communication and understanding, these SNOMED codes were converted to ICD10 codes by using the mapping tools mentioned above. The CHITRA database also allowed classification of data as diseases and comorbidities. Relevant outcomes were also extracted from both databases using customized scripts.

### Outcomes Measured

***Demographic details***: Age, length of stay (LOS), gender, and source of admission were tabulated and status at discharge from ICU was captured. The length of ICU stay for each ICD10 code was calculated as a difference between the date of admission and date of ICU discharge. The acute physiology scores, APACHE II (Acute Physiology and Chronic Health Evaluation II), were calculated from the acute physiological variables that were entered into the databases.^15^ The information collected and results generated were classified as pre-CHITRA and CHITRA data. Data cleaning, analysis, and tabulation were performed using the statistical software STATA (TM) v14.^16^ Admission diagnoses were tabulated using the ICD10 system. The most common disease codes were summarized and cross tabulated with respect to measured outcomes, from both pre-CHITRA and CHITRA databases.

### Statistical Analysis

The outcomes were summarized and cross tabulated for display. Analysis of continuous measure outcomes were compared using t test, and regression analyses were done with outcomes as dependent variables and time as the independent variable. The change in proportion of ICD10 classes over time was shown using a stacked area chart. Most frequent associations of comorbidities and diseases were displayed using the STATA command “tabplot” and represented as a heat map. As patients could have multiple overlapping comorbidities and diseases, cross tabulation would have not been appropriate to explore underlying associations between comorbidities, and between comorbidities and diseases. The most common comorbidities and associated diseases were subjected to correspondence analysis using the command “ca” in STATA(TM) v14.^16^ The R software package was used to generate rotational 3D plots after correspondence analysis.^17,18^

## RESULTS

The data in this study were extracted from clinical information systems used in the ICU for the past 12 years. From 2006 to 2014, data were extracted from the pre-CHITRA database and following this period, the CHITRA database was used. Table 1 shows demographics of all patients admitted to ICU and cross tabulated against clinical outcomes.

**Fig. 1:**
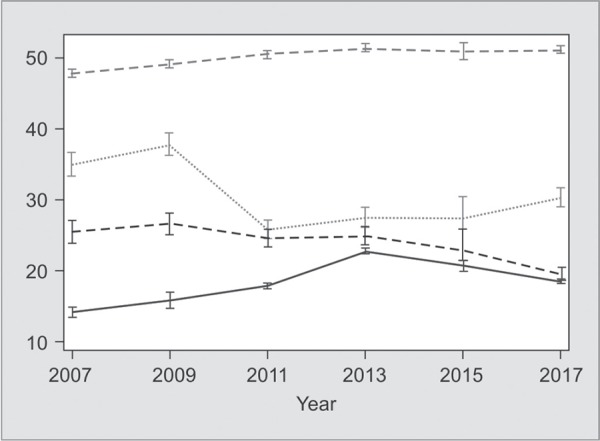
Trend of APACHE II and age of patients at admission and outcomes in ICU

A total of 18,940 patients were admitted with a male preponderance, and the median age of 52 years. Most of the admissions were from emergency room (62%), followed by inpatient wards (30%). Figure 1 shows the trends in age, APACHE score, and ICU mortality over the years. Regression analyses have shown that the age (coef. 0.30, *p* = 0.00, CI 0.23–0.38), and APACHE II scores (coef. 0.18, *p* = 0.000, CI 0.12–0.25) of patients had increased over the years. The ICU mortality had decreased (coef. 0.04, *p* = 0.000, CI -0.05– -0.03) over the years.

The most common acute admission diagnoses and corresponding ICD10 classification of disease codes from pre-CHITRA database are shown in Table 2. The two most common admission diagnoses which could be grouped together under an ICD10 class were, a) general symptoms and signs (ICD10 R50-R69) in 1,432 (10%) patients, and b) renal failure (ICD10 N17-19) in 1,412 (10%) patients. There were 573 admissions due to diseases of liver (ICD10 K70-K77) and this group had the highest mortality (41.01%).

Age in years, APACHE II, died and DAMA as percentage of admissions(%) are shown as mean and 95% confidence intervals, APACHE II: Acute Physiology and Chronic Health Evaluation II, DAMA: Discharge against medical advice, ICU: Intensive Care Unit.

**Table 1:** Demography

*Year (N)*	*Age years Median (IQR)*	*Gender M/F*	*Length of Stay days Median(IQR)*	*Outcome (%)*	*APACHE mean(sd)*
2006-8 (3905)	49 (33-62)	2509/1396	2 (1-3)	Alive (63) Died ICU (26) DAMA (11)	12.5 (6) 18.6 (7) 17.7 (5.7)
2009-11 (5869)	52 (35-65)	3740/2129	2 (1-3)	Alive (71) Died ICU (25) DAMA (4)	16.5 (7.4) 22 (8) 17 (5.5)
2012-14 (4258)	54 (37-65)	2733/1525	2 (1-4)	Alive (72) Died ICU (25) DAMA (3)	21 (8.6) 26 (8) 26 (8.5)
2015-18 (4908)	53 (36-65)	3038/1870	2 (1-5)	Alive (70) Died ICU (19) DAMA (11)	17.6 (7.3) 21.4 (7.5) 20.9 (7.4)
Total: 18940	52 (35-65)	12020/6920	2 (1-3)	Alive (70) Died ICU (23) DAMA (9)	18 (8.1) 23 (8.3) 21 (7.7)

APACHE II : Acute Physiology And Chronic Health Evaluation II, DAMA: Discharge against medical advice, IQR: Interquartile range, LOS: Length of stay in ICU, SD:standard deviation

**Table 2:** Pre-CHITRA (2006-2014) common diagnoses on admission

*Description (ICD10 Class)*	*N, (%)*	*APACHE Mean, (SD)*	*LOS Mean, (SD) days*	*Mortality: Died or DAMA (%)*
General symptoms and signs (R50-R69)	1432, (10%)	10.7, (11.1)	4.8, (6.3)	38
Renal failure (N17-N19)	1412, (10%)	10.5, (10.8)	4.48, (5.5)	30
Pneumonia due to bacteria (J13-J15.9)	1007, (7%)	11.8, (10.8)	5.5, (7.1)	30
External causes of morbidity (V01-X59)	852, (6%)	5.3, (8)	5.5, (6.9)	17
Hypertensive diseases (I10-I15)	835, (6%)	11.2, (10)	4.5, (6.4)	23
Endocrine diseases (E00-E35)	834, (6%)	12.8, (10.9)	4.7, (6.3)	25
Injury & poisons (S00-T14)	763, (5%)	5.9, (8.1)	5, (7.5)	15
Chronic lower respiratory diseases (J40-J47)	666, (5%)	9.9, (8.8)	4.9, (6.4)	21
Cerebrovascular diseases (I60-I69)	639, (4.5%)	10.2, (9.7)	4.9, (6)	23
Diseases of liver (K70-K77)	573, (4%)	11.8, (10.3)	3.8, (5.5)	41
Ischemic heart disease (I20-I25)	555, (4%)	12.6, (10.3)	4.2, (5.1)	29
Complications of surgical and medical care (T80-T88)	532, (4%)	4.9, (6.4)	3.4, (5)	17
Other disorders of genitourinary tract (N99)	394, (3%)	13.3, (12.5)	4, (4.5)	35
Infections of the skin and subcutaneous tissue (L00-L08)	298, (2%)	9.9, (10)	4.8, (6.1)	29

APACHE II: Acute Physiology And Chronic Health Evaluation II, DAMA: Discharge Against Medical Advice, ICU: Intensive care unit, ICD10: International Statistical Classification of Diseases and Related Health Problems 10th Revision, LOS: Length of stay in ICU. N: Numbers of diagnoses, %-in number of subjects, SD: Standard deviation

**Table 3:** Chitra (2015–2017) most common diagnosis

*Disease and ICD10 code*	*N (%)*	*LOS (hour), Median (IQR)*	*APACHE Mean (SD)*	*Mortality-Died and DAMA*
Type 1 respiratory failure (J96.90)	590 (12)	91 (47-180)	19.5 (8.1)	38%
Acute renal failure (N17.9)	570 (12)	74 (39-136)	22.5 (7.9)	43%
Pneumonia (J15.9, J18.9)	455 (9)	87 (45-177)	20 (7.5)	35%
Septic shock (R65.21)	448 (9)	75 (39-150)	20.6 (8.3)	52%
Sepsis (A41.9)	293 (6)	82 (47-160)	19.9 (7.9)	37%
Chronic kidney disease, unspecified (N18.9)	200 (4)	71 (43-132)	23.4 (8.3)	30%
Encephalopathy (G93.9)	180 (3.5)	90 (51-165)	20 (8.6)	38%
Urosepsis (N39.0)	157 (3)	70 (47-117)	20.8 (8.4)	26%
Non-ST elevation MI (I21.4)	152 (3)	80 (49-137)	22.8 (8.6)	33%
Febrile illness (R50.9)	126 (2.5)	65 (34-124)	18.7 (8)	16%

APACHE II: Acute Physiology And Chronic Health Evaluation II, DAMA: Discharge Against Medical Advice ICU: Intensive care unit, ICD10: International Statistical Classification of Diseases and Related Health Problems 10th Revision, LOS: Length of stay in ICU. N: Numbers of diagnoses, %-in number of subjects, SD: Standard deviation

Information from October 2015 to January 2018, which had been extracted from the CHITRA database, is shown in Table 3. Cross tabulations of the most common diseases with their severity of illness scores, corresponding mortalities, and length of stay have been displayed. Discharge against medical advice (DAMA) and death in ICU are shown as ICU mortality to provide more conservative estimates of ICU survival. Type 1 respiratory failure (ICD10 J96.90) was most common diagnosis made at admission. It was followed by acute renal failure and pneumonia. Septic shock (ICD 10 R65.21) had highest mortality (52%) in the CHITRA dataset.

**Fig. 2:**
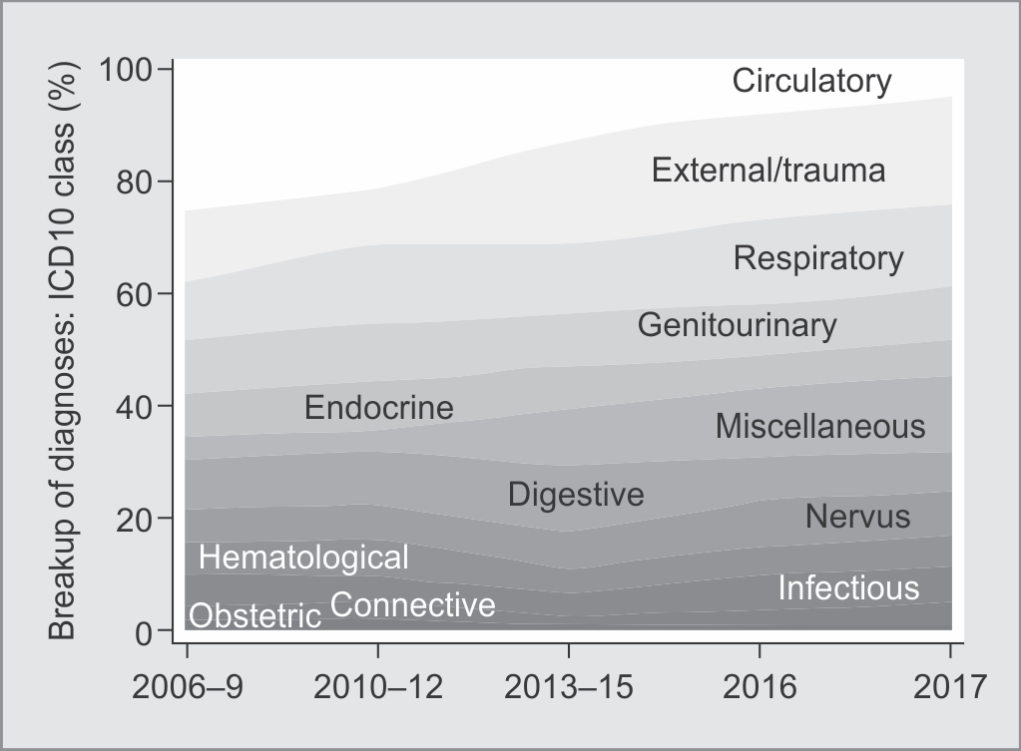
Stacked area chart, displays secular trends in ICD10 classes at admission over 12 years

There are 22 ICD10 classes, and all are not relevant in adult ICUs. For ease of visual display, they have been collapsed into 12 mutually exclusive classes. The variation in numbers of diagnoses in these ICD10 classes over time has been shown as a percentage of the total number of diagnoses in the stacked area chart in Figure 2. The secular trends in ICD10 diagnostic classes at admission for the last 12 years can be appreciated by the variation in the areas occupied by each ICD10 class. The increase in number of external causes of morbidity/trauma and miscellaneous diagnoses can be appreciated by the increase in area occupied by these disease classes over time.

**Fig. 3:**
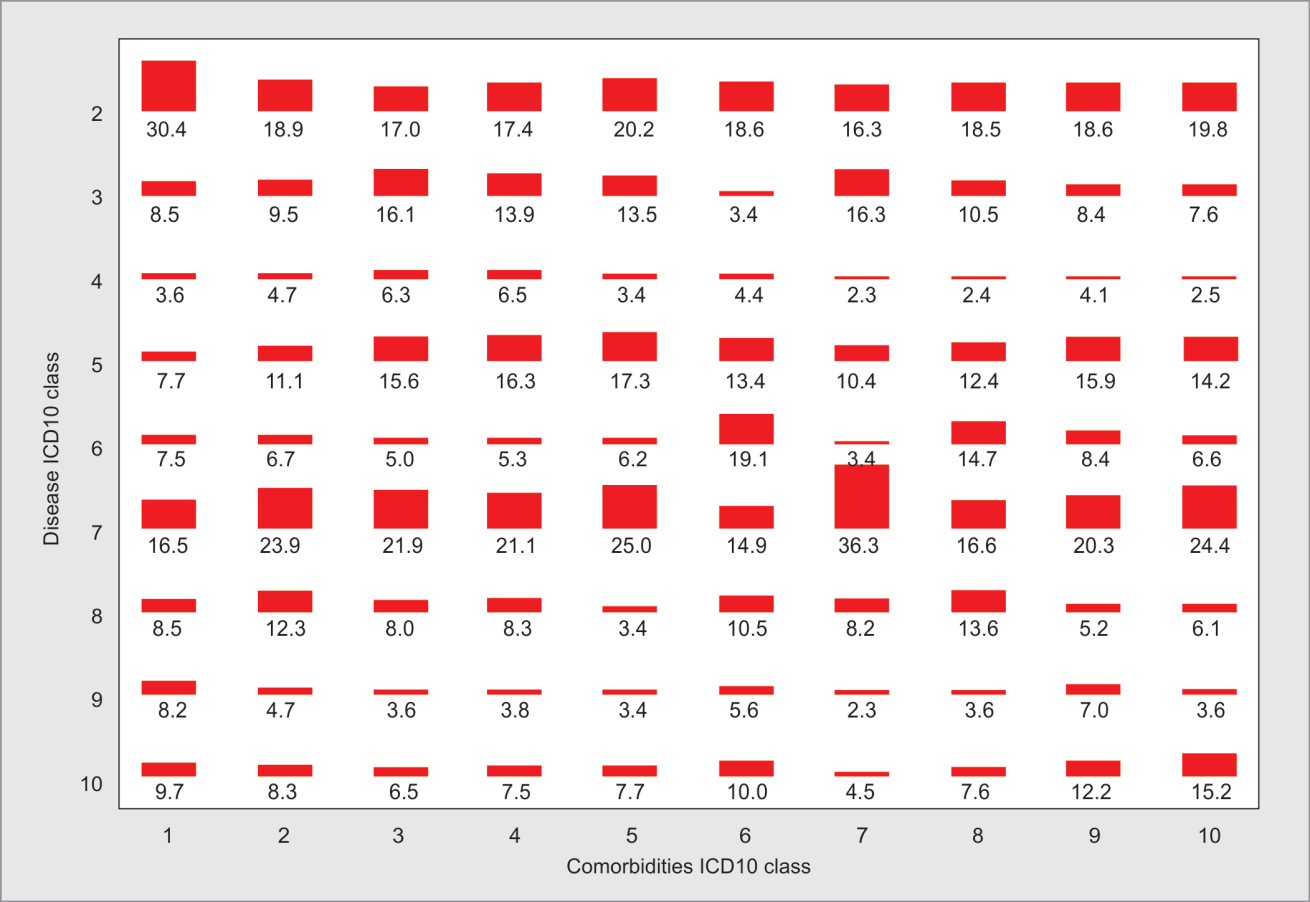
Heat map of comorbidities vs diseases

From 2015 onwards in the CHITRA database, comorbidities and admission diagnoses were captured as separate entities. There were 12,691 diagnoses from 4,908 patients of which 3,552 (28%) were recorded as comorbidities and 9,139 (72%) were recorded as diseases. There was an average of four diseases (range 1–15) entered as diagnoses for each patient. There was an average of one comorbidity recorded for each patient (range 0–10), and 30% of patients did not have any comorbidity recorded. Circulatory system (hypertension and heart disease) comorbidities and endocrine (diabetes mellitus) comorbidities comprised more than 80% of the recorded comorbidities. As each patient could have many comorbidities and diseases, the commonly encountered associations between comorbidities and diseases are displayed in Figure 3. The most common comorbidities are shown as columns and the disease percentages for each column are shown as a heat map in the Figure 3. The shaded area is proportional to the figures in the columns. Trauma, miscellaneous, and toxicology were major associated diseases (30%) in patients without any comorbidities. Patients with respiratory system related comorbidities had increased admission with an acute diagnosis from respiratory disease (36%).

Circulatory: ICD10 I, External Causes of morbidity/Trauma: ICD10 S-T, V-Y, Respiratory: ICD10 J, Genito_Urinary: ICD10 N, Endocrine: ICD 10 E, Miscellaneous: ICD10 R (Not elsewhere classified), Digestive: ICD10 K, Nervous: ICD10 F-G, Hematological: ICD10 C-D, Infectious: ICD10A-B, Connective: ICD10 L-M, Obstetric: ICD10 O-P

**Fig. 4:**
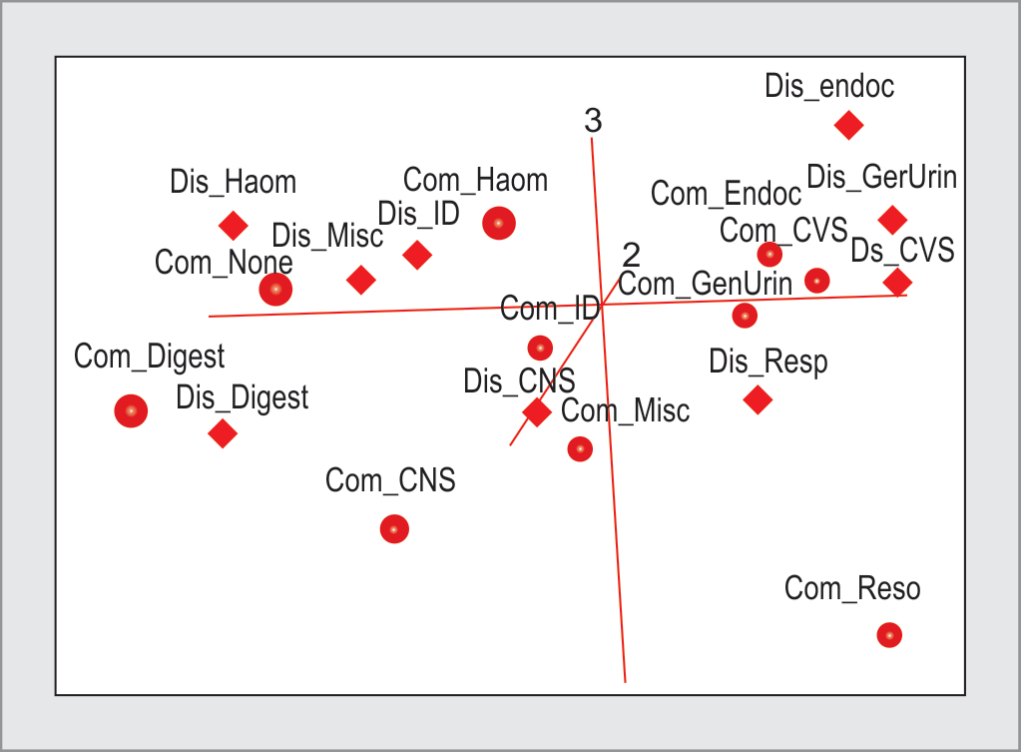
Three-dimensional plot of correspondence analysis comorbidities and diseases

A three-dimensional (3D) rotational plot of a correspondence analysis of comorbidities and diseases is shown in Figure 4 in two dimensions. These three dimensions could visualize 90% of the variability of the data. The 3D plots were rotated to optimize visualizations and associations. Diseases and comorbidities clustered close to the intersection of the three axes are not strongly associated. Digestive comorbidities and diseases are clustered close to each other in one axis and far away from the intersection displaying the strong association. Cardiovascular, endocrine and genitourinary comorbidities and diseases were expectedly clustered together along an axis. The respiratory comorbidities were not associated with any other disease other than respiratory disease. No comorbidities and miscellaneous diseases were associated, but this is not well seen in the projection in Figure 4.

1: No Comorbidity, 2: Miscellaneous external disease (ICD10 H, L, M, Q-R, S-Z), 3: Circulatory (ICD10 I), 4: Endocrine (ICD10 E), 5: Genitourinary (ICD10 N), 6: Digestive (ICD10 K), 7: Respiratory (ICD10 J), 8: Nervous (ICD10 F-G), 9: Hematological-Neoplastic (ICD10 C-D), 10: Infectious and parasitic (ICD10 A-B)

Com, comorbidity; Dis, disease; None, no comorbidity; Misc, miscellaneous external disease (ICD10 H, L, M, Q-R, S-Z); CVS, circulatory (ICD10 I); Endoc, endocrine (ICD10 E); GenUrin, genito-urinary (ICD10 N); Digest, digestive (ICD10 K); Resp, respiratory (ICD10 J); CNS, nervous (ICD10 F-G); Hem, haematological-neoplastic (ICD10 C-D); ID, infectious and parasitic (ICD10 A-B)

## DISCUSSION

This study has captured epidemiological trends over a 12-year period from a single center. The ICU continued to be a closed ICU since 2006 and there were no major changes in organizational structure and no specialized units were introduced. Data from over 18,000 patients have been tabulated with key outcome parameters and disease codes, which could serve as a reference point for future comparisons for Indian ICUs. This study has also shown the utility of the CHITRA system in capturing epidemiological data.

The median age at admission to ICU in our study (52 years) differed from ICON audit (62 years) and KIND study (64.3 years), which is not unexpected as developing countries have younger populations.^19,20^ Of the total admissions, male patients were higher in number with male to female ratio of 1.73:1. Similar higher male to female admissions were shown from Korean, Austrian, and American ICUs.^20–22^ In this study, most of the admissions were from emergency room followed by inpatient wards, similar to the data from American ICUs.^22^

Studies from Korea, Austria, and Scandinavia have reported mortality rates in ICU of 13.8%, 17.6%, and 16.9%, respectively.^20,21,23^ The mortality over our study period was higher than these studies (23%). This is even higher when compared to data from INDICAPS (18.1%) and ICON audit (14%).^7,19^ Average APACHE II of patients who survived ICU in our study was 18, and who did not survive ICU was 23. This is comparable to the INDICAPS study where the mean APACHE II at admission of survivors was 16.1 and non survivors was 23.6.^7^ The dissimilarity in mortality noted between our study and INDICAPS study may be due to differences in health care system across the countries.

In a developing country, financial constraint may compel family members to opt for discharging patients against medical advice (DAMA). The APACHE II scores for these DAMA patients is similar to the patients who died in ICU suggesting that severity of illness and perceived poor prognosis may have influenced this decision. In our study, there was a trend towards decrease in ICU mortality over the years despite an increase in age and APACHE II score which may reflect improvement in general standards of critical care over the last decade. This is supported by published data from the same institution in 1998, where the average age was 42 years, and the ICU mortality rate was 38%.^24^

Length of stay in ICU remained unchanged around 2 days over the study period. ICON audit showed mean LOS of 3 days.^19^ Data form US has shown significant decrease in mortality (from 5.81% to 5.7%) and length of ICU stay (from 3.11 to 3.0 days) over 5 years (2009 to 2013).^25^

From pre-CHITRA database (Table 2), largest admission diagnosis group was with general symptoms and signs (ICD9- R50-69) which could include fever, hemorrhage, shock, and other unspecified illness. The next common diagnostic group i.e. renal failure (ICD9- N17-19, 10%), was followed by pneumonia (ICD9- J13-15.9, 7%) and accidents (ICD9 -V01-X59, 6%). This contrasts with the data from developed countries which show cardiovascular diseases, trauma, surgery, and surgery unspecified as most frequent reasons for ICU admissions.^21^ The multicenter point prevalence study of infection, EPIC II, has shown that pneumonia was the most common diagnosis among infections.^26^ It is difficult to draw conclusions from data which were collected a decade ago as the standards for documentation of diagnoses would have varied. From 2015, the CHITRA database was used for data collection, and the inbuilt mapping of text to SNOMED probably improved the accuracy of classification. This data (Table 3), shows respiratory failure as the most common reason for ICU admission, followed by acute renal failure, septic shock, and pneumonia. The INDICAPS study had shown diseases involving the cardiovascular system as the most common reason for ICU admission to medical ICU.^7^

The stacked area chart (Fig. 2) shows a general decrease in proportion of circulatory system diseases and endocrine diseases over the study period. Also, there was an observed increase in proportion of trauma and miscellaneous (toxicology) diseases. Five-year trends in US had shown decrease in both cardiovascular and toxicology diseases with increase in endocrine disorders, especially diabetes related complications.^25^

Our observations reflect changing case-mix and demographics of admitted patients. As expected, patients without any comorbidities had miscellaneous, trauma, and toxin related admissions, as compared to other acute admission diagnosis. The 3D plotting of the correspondence analysis revealed interesting associations, which was not shown in standard cross tabulations. A report from Spanish health survey gives a comprehensive explanation of correspondence analysis applied in the context of nation's state of health.^27^ By visualizing the table in the form of a map of points representing the rows and columns of the table, it has shown the use of correspondence analysis to interpret a simple cross tabulation.^27^ The CHITRA application has allowed comorbidities and diseases to be accurately coded and captured. To the best of our knowledge, 3D plotting of a correspondence analysis between ICU comorbidities and diseases has not been commonly reported. Clinical information systems deployed in the ICU will continue to generate multi-dimensional data and 3D plots are part of the toolkits for multivariate data analysis.^28^

Our study had a number of limitations. The diagnoses tabulated are from those recorded at admission and are not confirmed diagnoses. The diagnoses have been abstracted from records, and in the earlier years, there was no rigor in documentation. Metrics tabulated are indicative of outcomes, considering the presence of at least that disease as diagnosis. Presence of additional diagnosis may have had an unfavorable impact on outcomes. An outcome like 90-day mortality would have been more appropriate but was logically difficult to capture. The most common disease groupings have been displayed. Efficient visual presentation of all combinations of diseases and outcomes, from such a large group of patients, and diagnoses is difficult.

The goal of creating national datasets will be served if more ICUs engage in systematic collection and collation of epidemiological data. Local and national level diagnostic, procedural and outcome data has to be captured to optimize health care delivery. Accurate collection, storage, and analysis of this high dimensional data will need planning of information technology resources. The CHITRA system can be adapted and effectively utilized for further multicenter epidemiological studies in the future.

## CONCLUSION

Intensive care units are data rich environments and collection and analysis of this high dimensional data will require adoption of new technologies. This study has shown the utility of the CHITRA system, which was developed by the ISCCM in capturing and storing epidemiological data in a systematic manner.
